# Comparative analysis of optional hunting behavior in Cricetinae hamsters using the data compression approach

**DOI:** 10.1186/s12983-024-00540-4

**Published:** 2024-07-15

**Authors:** J. Levenets, S. Panteleeva, Zh. Reznikova, A. Gureeva, V. Kupriyanov, N. Feoktistova, A. Surov

**Affiliations:** 1 Institute of Systematics and Ecology of Animals, Siberian Branch, Russian Academy of Sciences, Novosibirsk, 630091 Russia; 2 Novosibirsk State University, Novosibirsk, 630090 Russia; 3 A.N. Severtsov Institute of Ecology and Evolution, Russian Academy of Sciences, Moscow, 119071 Russia

**Keywords:** Hamsters, Hunting, Behavioral sequences, Evolution, Data compression, Homogeneity

## Abstract

Research into the hunting behavior in members of the Cricetidae family offers an opportunity to reveal what changes in the predatory behavioral sequences occur when a rodent species shifts from an omnivorous to a predatory lifestyle. The study tests the following hypotheses: are there phylogenetic differences in the divergence of species’ predatory lifestyles in hamsters or do ecological factors lead to shaping their hunting behavior? We applied the data compression approach for performing comparative analysis of hunting patterns as biological “texts.” The study presents a comparative analysis of hunting behaviors in five Cricetinae species, focusing on the new data obtained for the desert hamster *Phodopus roborovskii* whose behavior has never been studied before. The hunting behavior of *P. roborovskii* appeared to be the most variable one. In contrast, behavioral sequences in *P. campbelli* and *Allocricetulus curtatus* display more significant order and predictability of behavior during hunting. Optional hunting behavior in the most ancient species *P. roborovskii* displayed similarities with obligate patterns in “young” *Allocricetulus s*pecies. It thus turned out to be the most advanced hunter among members of the *Phodopus* genus. Differences in hunting sequences among *Phodopus* representatives suggest that the hunting behavior of these species, despite its optional mode, was subject to selection during species splitting within the genus. These results did not reveal the role played by phylogenetic differences in the divergence of species’ predatory lifestyles. They suggested that ecological conditions are the main factors in speciation of the hunting behavior in hamsters.

**Supplementary Information:**

The online version contains supplementary material available at 10.1186/s12983-024-00540-4.

Most rodents feed on a combination of vegetation, seeds, and animal matters, and all of them can be considered omnivorous to some degree [1]. Displays of hunting behavior in rodents vary from highly specialized predation in carnivorous grasshopper mice [2–6] to skillful but optional insect hunting in species with different types of diet [7–9]. Recent studies consider carnivorous rodents to be specialists in eating other animals, mainly invertebrates, which have evolved adaptations enhancing prey's detection, capture, and consumption. An insectivorous diet is regarded as a subcategory under carnivory [10–12]. In particular, a comprehensive phylogenetic analysis of carnivorous murids of the Indo-Australian Archipelago demonstrated that carnivory evolved independently in each biogeographic unit. The origin of carnivory was followed by evolution of more specialized carnivorous ecomorphs such as vermivores, insectivores, and amphibious rats [10].

It is of distinct interest what changes in the predatory behavioral sequences occur when a rodent species shifts from an omnivorous to a predatory lifestyle, including insect hunting. For identifying specific changes, related carnivorous/insectivorous and those omnivorous species that display different levels of optional hunting behavior need to be compared. Members of the Cricetidae family offer an opportunity to make the required kind of comparisons, including comparison of the carnivorous southern and northern grasshopper mice mentioned above [3, 13] and species that display hunting behavior to a greater (golden hamster (*Mesocricetus auratus*, Waterhouse, 1839) [2, 14], Tuva silver vole (*Alticola tuvinicus*, Ognev, 1950) [15]) or lesser extent (hispid cotton rat (*Sigmodon hispidus*, Say and Ord, 1825) [7], narrow-headed vole (*Lasiopodomys gregalis*, Pallas, 1779) [15]). Langley [16] has recently compared the predatory attack of specialized carnivorous southern and northern grasshopper mice (*Onychomys torridus*, Coues, 1874; *O. leucogaster*, Wied-Neuwied, 1841) with that of the deer mouse (*Peromyscus maniculatus*, Wagner, 1845) and found specific differences in their hunting behavioral patterns: the use of forepaws to capture invertebrate prey in predatory hamsters, obligate display of hunting patterns in specialized species and facultative hunting behavior requiring experience in omnivorous rodents, less delay during an attack and resistance to attack inhibition in *Onychomys*. Seizing prey with the forepaws is a more progressive evolutionary trait of specialized predators, such as felines, whereas insectivores, such as shrews, display primitive attacks with a series of bites [17]. Langley [3] showed that in rodents, prey capture appears as a progressive trait, with specialized grasshopper mice displaying the most advanced pattern. It is worth noting that these rodents are members of the same *Peromyscine* clade, and fossil evidence shows that grasshopper mice have evolved from omnivorous ancestors [18] (Carleton and Eshelman, 1979). Analysis of the phylogenetic and environmental components in the development of predation in related species can provide a framework for describing the evolution of a carnivorous lifestyle in rodents and other mammals. However, most studies have been done on North and South American species, while the Eurasian continent still needs to be explored.

We suggest that Cricetinae hamsters include species with a pronounced predatory behavior similar to that of deer mice among Palaearctic hamsters. The niche of Eversmann’s hamster (*Allocricetulus eversmanni*, Brandt, 1859) in the prairies of North America is occupied by *O. leucogaster*, while *O. torridus* from the Nearctic deserts resembles the gray dwarf hamster (*Cricetulus migratorius*, Pallas, 1773) in appearance [19]. In our view, *O. torridus* is similar in many respects to desert hamster (*Phodopus roborovskii*, Satunin, 1903). Vorontsov [20] investigated the morphology of the digestive system and stomach contents and found that Palaearctic hamsters display a mixed diet with wide use of plants’ generative and vegetative parts: for *Phodopus* and *Allocricetulus*, animal-protein food is a necessary component of their diet. Insects are part of the diet of the Djungarian hamster (*Phodopus sungorus*, Pallas, 1773), Campbell’s dwarf hamster (*P. campbelli*, Thomas, 1905) and *P. roborovskii* [21]. In addition to insects, invertebrates and small vertebrates constitute a vital part of the diet of *A. eversmanni* and Mongolian hamsters [19]. Species vary in habitat preferences [22, 23]. *A. eversmanni* and *P. sungorus* inhabit lowland steppes and semi-deserts, while the habitats of *A. curtatus* and *P. roborovskii* are limited to fixed sands and saltwort semi-deserts. *P. Campbelli* inhabits steppes and semi-deserts of Central Asia, overlapping with the ranges of *A. curtatus* and *P. roborovski* in the border area of Central Mongolia and Northern China.

*Phodopus* is one of the most ancient groups in the subfamily Cricetinae (the divergence time is appr. 8.5–12.2 MY) [24]. *P. roborovskii* diverged from a common branch about 5–5.7 MY ago, while the divergence of *P. sungorus* and Campbell’s dwarf hamster (*P. campbelli*, Thomas, 1905) dates back to 0.8–1 MY [24, 25]. Separation of the genus *Allocricetulus* occurred in the early Pliocene (about 3.6–5.3 MY), and the divergence of species within the genus occurred 0.3 MY ago [22, 25].

Among all these Cricetinae species, the behavioral patterns of insect hunting have been studied only in four hamster species, from the simplest variant in *P. sungorus* and *P. campbelli*, to the more specialized in *A. eversmanni* and *A. curtatus* [26]: in both *Allocricetulus* species, hunting behavior was obligatorily displayed in all individuals, combined with the ability to initiate an attack by seizing with the paws, which is a sign of specialized hunting behavior [16]. The mathematical analysis revealed the quantitative behavioral similarities between the latter two species and the generalized predator Norway rat (*Rattus norvegicus*, Berkenhout, 1769), possibly caused by their particular abilities to manipulate forepaws when handling the prey [27].

In this study, we investigated behavioral patterns in non-predatory Cricetinae with different degrees of hunting behavior: obligate in *Allocricetulus* and facultative in *Phodopus*. Here, we use the data compression approach [28, 29] and quantitative relations of behavioral elements to perform a comparative analysis of hunting behaviors in five Cricetinae species focusing on the new data obtained on the desert hamster *P. roborovskii* whose behavior has never studied before. Some quantitative characteristics do not allow a clear classification and assessment of the proximity of behavioral tuplets. Therefore, we use these data by superimposing them on a statistically supported apparatus of data compression methods: assessing complexity (variability) and homogeneity (proximity) at the level of whole behavioral tuplets. In this way, we can answer the question of which elements are responsible for variation in behavior and compare possible evolutionary trajectories based on the structural characteristics of hunting sequences. We attempted to reveal the possible role of phylogenetic differences in the divergence of species’ predatory lifestyles in hamsters. Alternatively, ecological factors can play a leading role in shaping the hunting behavior of hamsters. We cannot answer what environmental factors have contributed to the divergence of predatory lifestyles without conducting studies in natural conditions. However, based on general ideas about rodents’ behavioral evolution mechanisms [16], we can attempt alternatively to exclude historical evolutionary or environmental factors. By analyzing the sequences of actions spontaneously exhibited by naïve animals in the laboratory, we try to assess the degree of similarity in the innate basis of hunting behavior. If differences in the structure of hunting behavior are due to a phylogenetic factors, then the comparative data obtained will be consistent with molecular genetic data. Otherwise, one can assume the leading role of environmental factors, that is, adaptive radiation of Cricetinae.

## Materials and methods

### Animals and housing

Five species belonging to the subfamily Cricetinae were used in the study: desert hamster (*P. roborovskii*), Campbell’s dwarf hamster (*P. campbelli*), Djungarian hamster (*P. sungorus*), Eversmann’s hamster (*A. eversmanni*), and Mongolian hamster (*A. curtatus*) (Table 1).

**Table 1:** The material studied

Species	Number of animals	Number of tests
Desert hamster (*P.* *roborovskii*)	♂6 / 8♀	42
Campbell’s dwarf hamster (*P. campbelli*)	♂11 / 8♀	133
Djungarian hamster (*P. sungorus*)	♂16 / 14♀	90
Eversmann’s hamster (*A. eversmanni*)	♂1 / 7♀	24
Mongolian hamster (*A. curtatus*)	♂5 / 8♀	39

In nature, the Djungarian hamster is confined to the true herb bunchgrass and bunchgrass steppe zones, whereas the Campbell’s hamster primarily inhabits the dry steppe and semi-desert zones [21]. The desert hamster *P. roborovskii* inhabits deserts and semi-deserts and is confined to sands. The ranges of the latter two species partially overlap, but they are not synbiotopic [21, 30, 31]. This overlapping is observed in the areas where sand biotopes invade the natural zones of semi-deserts and dry steppes. The Eversmann’s hamster inhabits lowland steppes and semi-deserts. The Mongolian hamster is confined to fixed sands and saltwort semi-deserts [32]. Ecologically, the Mongolian and desert hamsters are very close and occur together in different parts of the range. However, the differences in size, population density, etc. is likely to prevent direct competition.

In general, the type of diet of all the investigated species is mixed and based on seeds, vegetative parts of plants, and protein food. Depending on the season, the dominant foodstuffs replace each other [19, 21]. Analysis of stomach contents shows that the diet of *P. roborovskii* consisted of 47% seeds, 42% animal-protein food (mainly insects) and 11% green plant parts (Tuva, August 1987, *n* = 7). In *P. sungorus*, respectively, 20% / 15% / 65% (Khakassia, July 1989, *n* = 5); in *P. campbelli*, 36% / 11% / 53% (Tuva, August 1987, *n* = 8) [21]. In *A. eversmanni* – 25 seeds 43%, animal-protein food 32%, vegetative parts of plants 25%. The contents of the cheek pouches of *A. curtatus* include of 73.7% animal-protein food and only 26.5% plant food [19].

Using the same data (behavioral sequences) obtained in a previous study for both *Allocricetulus* and *P. campbelli* species [26], the data sample for *P. sungorus* was expanded from 13 to 30 animals. Data on the hunting behavior of *P. roborovskii* were obtained for the first time. Therefore, the quantification data for *Allocricetulus* and *P. campbelli* are the same as previously published studies by Levenets et al. [26]. However, in this study, we use them to explain the new data obtained by the data compression method. Data compression operates on the structural characteristics of 'texts' at the level of whole sequences and provides insight into what regularities occur in hunting behavior. To uncover these regularities, we estimate quantitative relations of behavioral elements.

The study of *Allocricetulus* hamsters and *P. campbelli* was carried out in 2019–2022 at the Tchernogolovka Biological Station, Severtsov Institute of Ecology and Evolution, Russian Academy of Sciences, using animals from the joint use center Living Collection of Mammalian Wildlife Species. Outbred *P. campbelli* and *P. sungorus* were studied in the vivarium of the Institute of Animal Systematics and Ecology, Siberian Branch, Russian Academy of Sciences, in 2016–2018.

The animals were the offspring of wild ones, later bred in captivity for 10–20 generations. All animals were mature, under the age of two years, and had been born in vivarium. Before the experiment, they did not encounter potential prey and did not have any hunting experience.

All the animals were housed in plastic cages containing cotton nesting material under a 16:8 light/dark cycle at 23–26 °C. They were fed each day once with mixed seeds, dried shrimps, cottage cheese, and boiled chicken, and had ad libitum access to water; all animals were provided with food before being taken into experimental arenas (see details in [9]). We used imago and final instar nymphs of the lobster cockroach (*Nauphoeta cinerea,* Olivier, 1789) (27.93 ± 0.40 mm) as live mobile prey.

### Experimental procedures and data analysis

Similarly to [9, 15, 33], we placed each rodent in a separate plastic arena (30 × 30 × 35 cm). In each trial, an insect was placed into the arena manually, 5 min after the rodent. Video recordings were made using a Sony HDR-AS200V (60 frames per second). After each test, the arena was cleaned using 70% alcohol. Each animal received up to three insects in turn during each of three test sessions.

Spontaneous reactions to live insects were recorded. Three test sessions were conducted with each species, excluding *P. campbelli* and *P. sungorus* (up to seven test sessions). The reason for more repeated tests is that the frequency of hunting behavior in these species was lower than in the other hamsters studied. The first three trials in which the animal showed hunting behavior were selected for the analysis; after that, the repetition of the trials was stopped. Thus, the number of hunting behavior occurrences for all species tested was equivalent.

We used the previously developed alphabet of 17 letters to analyze hunting behaviors by assigning a letter to each behavioral element. Using the Noldus Observer XT behavioral coding software and the alphabet consisting of behavioral elements in the order of their appearance, without taking into account their duration, we obtained behavioral sequences (or, in our case, “hunting sequence,” for brevity, “sequence”). Note that “hunting sequence” can be considered the case of “behavioral tuplet,” which is an umbrella term for “a stable and recurrent change of behavioral elements” [33] (see also [34]). All sequences obtained were exported into text files (format.txt), each file for each of the five species, with sequences being blank-separated in each file.

Based on the frequency of occurrence in the sequences, the obtained 17 behavioral elements were divided into three groups. The first one included “key” elements that are strongly necessary for accomplishing the hunting, such as bite (W) and seizing an insect with paws (E). These elements have always been present in hunting sequences, which ended with successful catching and eating of prey. The second group included “auxiliary” elements related to prey handling (R), sniffing (D), pursuing the prey by walking (S) or running (Q), carrying the prey in the teeth (G), and nibbling the insect’s legs (H). The third group consisted of the “noise” elements which did not influence the performance of the sequences at all: (C) freezing, (V) 90° body turn, (B) U-turn, (F) turning of the head, (Y) rearing against the wall, (I) free-standing rearing, (U) backward movement, (X) self-grooming, and (J) jump. Note that the “noise” and “auxiliary” elements were not present in all the sequences.

Only cases of successful hunting were analyzed. We call “successful” all the cases of hunting that ended with catching the prey (even after several false attacks), while referring to the rest of the situations as “unsuccessful” (the animal stopped actively seeking and pursuing prey). Note that an animal could manifest its attempts to hunt during subsequent tests, and not from the very first test. We evaluated hunting success as the ratio between the number of sequences ending in catching prey and unsuccessful ones and the number of different behavioral elements per sequence – “length.” We compared ‘hunting rates’ in studied species, that is, relations between the length of sequences (expressed in the number of elements) and their duration in seconds. The duration of the hunt was measured from the appearance of the first element in the sequence and ended on the element preceding consumption. Beginning of an attack was considered to be associated with the element “bite” (W) or “seizing with paws” (E) when either of these characters occurred first in the sequence or followed elements “S” or “Q” (pursuit of prey) and “D” (sniffing).

We applied two mathematical methods to compare hunting behavior in five hamster species. The data compression method based on the ideas of Kolmogorov complexity and on using data compressors [28] allows one to compare behavioral patterns as “texts” and to evaluate their flexibility and succinctness. This approach is based on the ability of archiver programs to find regularities in any “text,” that is, any characteristic of a text that makes it more predictable, such as frequency of occurrence of letters and subsequences and so on. To compare the organizational complexity of species-specific hunting behavior, we represent the sequence of symbols as text files. These text files should then be compressed by the chosen data compression method. The level of compression corresponds to the ratio between the length of the file after and before the compression. The difference between the compression ratios of files to be compared reflects the difference between the complexities of the symbol sequences recorded. Therefore, we can use the compression ratio as a species-specific characteristic of complexity.

However, the hunting modes could differ in different species. The differences concern the order of particular behavioral elements, as well as some aspects of hunting attacks. This means that although different rodent species display similar predictability of transitions between elements within sequences, and thus similar levels of complexity, they possibly possess the different structure of hunting behavior. We applied the compression-based method for homogeneity testing as a new tool to evaluate differences between the structural features of the ethological “texts” [27]. We tested the hypothesis whether the behavioral sequences of different species as “texts” are generated either by a single source or by different ones. The main idea of the approach is to combine fragments of the behavioral sequence of one species (“text X”) with fragments of another one (“text Y”), and then compress the combined sequences by an archiver. The text files containing similar sequences will be compressed better. Data of pairwise comparisons of two “texts” are presented as a 2 × 2 matrix. The degree of proximity is expressed as the association coefficient for the resulting matrices [35]. All the produced association coefficients are placed in an n-dimensional symmetric matrix. Based on the matrix with association coefficients, we performed a joining cluster analysis (tree clustering) using Euclidean distance as a metric. The free software PAST (PAleontological STatistics) v. 3.25 was used for clustering.

For both methods, we applied the open-source data compressor 7-zip v. 19.00 (64-bit), which uses the algorithm of data compression called Bzip2 (compressed file format.bz2). The following parameters were used in the graphical user interface (GUI) for archiving: Compression level normal; dictionary size, 100 kb; and the number of CPU threads, 6.

We used the Fisher’s exact test (the free software “R” v.3.6.0) to compare the proportions of successful and unsuccessful stereotypes in different species, the fractions of different types of behavioral elements as well as the difference between the 2 × 2 matrix obtained when comparing the sequences (see details in Supplementary).

The Kruskal–Wallis *H* test was used to compare hunting sequence lengths, the hunting rates, and the number of individual behavioral elements. A matrix of transition probabilities from one behavioral element to another was calculated to construct the scheme of hunting (see [26, 36]). The arrows on the scheme show the probabilities of transition from one element to another.

We used the Mann–Whitney–Wilcoxon *U*-test for pairwise comparisons of the compression ratio. Data are expressed as median, range and first and third quartiles. Based on the association coefficients (see details below), we performed joining cluster analysis (tree clustering) using Euclidean distance as a metric. The free software PAST (PAleontological STatistics) v. 3.25 was used for clustering, *U*- and *H*- test.

## Results

### Comparison of behavioral patterns

In this section, we combine and analyze together the new data on the desert hamster with the partially published and supplemented data on the other four species in order to complete the whole picture of their hunting behavior.

In our experiments, 9 out 14 (64.3%) *P. roborovskii*, 12 out of 19 (63.2%) *P. campbelli*, 12 out of 30 (40.0%) *P. sungorus*, all 8 (100%) *A. eversmanni*, and all 13 (100%) *A. curtatus* individuals demonstrated a complete hunting sequence that ended with killing the prey.

In all the tests, the hunting success rates of *P. roborovskii* (56%, 39 of 70), *P. campbelli* (51%, 43 of 85), and *A. eversmanni* (46%, 60 of 129) hamsters did not differ from each other and were lower than those of *A. curtatus* (81%, 115 of 142) and *P. sungorus* (70%, 76 of 109) (Fisher’s exact test with Bonferroni correction, *p* < 0.001 for all cases).

The length of the hunting behavior sequences, the hunting rate, and the number of individual behavior elements did not differ between the first, second, and third tests. The sequence length was similar in all species, except for *P. roborovskii*, in which this parameter was significantly higher. The hunting rate of all the studied species, except for *P. campbelli*, did not differ significantly. The total number of behavioral patterns “biting” (W) and “pawing” (E) in the hunting sequences of *P. roborovskii* was greater than that in the other species. *P. roborovskii* displayed numbers of prey handling (“R”) similar to those for *Allocricetulus* hamsters and differed from those for other *Phodopus* species (Table 2).

**Table 2:** Parameters of the hunting behavior in studied species

	**Species**				
	***P. roborovskii***	***P. campbelli***	***P. sungorus***	***A. eversmanni***	***A. curtatus***
Sequence length (number of elements)	62 (36–92)^**b**^	28 (16–46)^**a**^	16 (12–22)^**a**^	20 (14–30)^**a**^	21 (12–30)^**a**^
Hunting rate (elements per sec)	3 (2.5–3.8)^**a**^	1.4 (1–1.9)^**b**^	2.6 (2.1–3.5)^**a**^	2.3 (1.9–2.7)^**a**^	3 (2.4–3.6)^**a**^
Number of “Bites” (W)	17 (12.5–26.5)^**c**^	8 (4.5–14)^**b**^	4 (2–10)^**a**^	5.5 (3–9)^**a**^	6 (3–8)^**a**^
Number of “Seizing with paws” (E)	25 (16–36.5)^**c**^	10 (5.5–18)^**b**^	6 (2–10)^**a**^	6 (3–9)^**a**^	6 (3–11)^**a**^
Number of “Prey handling” (R)	7 (4–10)^**a**^	1 (0–2)^**b**^	2 (2–10)^**b**^	5 (3–7)^**a**^	6 (4–8)^**a**^
Number of “Nibbling insects’ legs” (H)	1 (0.5–4)^**a**^	3 (2–6)^**b**^	0 (2–10)^**a**^	1 (0–2)^**a**^	1 (0–1)^**a**^

The median and the first and third quartiles are presented: Me (Q1–Q3). Data marked with the same characters **a**, **b**, and **c** within the same line are not significantly different (*H*-test with Bonferroni correction, *p* < 0.0003 for all cases)

**Fig. 1:**
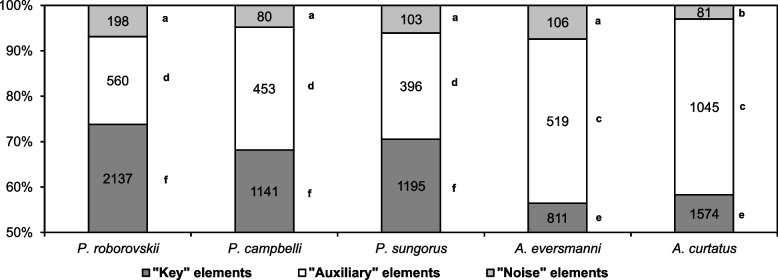
The proportion of different groups of behavioral elements in the studied species. Numbers indicate the number of elements. The same letters (**a**, **b**, **c**, **d**, **e**, **f**) indicate no significant difference between the same types of behavioral elements (‘Key’, ‘Auxiliary’, ‘Noise’) in different species (*p* < 0.0014) according to Fisher’s exact test with Bonferroni correction

The proportions of different behavioral elements in sequences varied. The largest proportion of key elements was detected in hunting sequences in *Phodopus*, and that of auxiliary elements was observed in sequences in *Allocricetulus* (Fig. 1). The proportion of noise elements in hunting sequences was lower in *A. curtatus* than in other studied species.

**Fig. 2:**
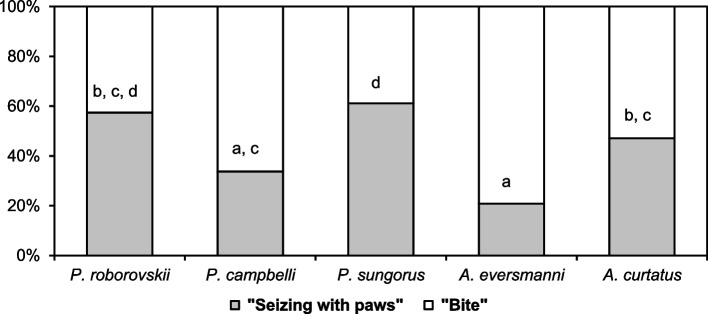
The ratio between the number of attacks that began with seizing with the forepaws and teeth and attacks that began with a bite. Data marked with the same characters **a**, **b**, and **c** are not significantly different (Fisher’s exact test with Bonferroni correction, *p* < 0.0003 for all cases)

All the studied hamsters were able to start attacking the insect both with their teeth (element “W” – bite) and forepaws (“E” – seizing with paws). Hamsters bit and seized prey with their forepaws not only at the beginning of an attack: the animals could repeat bites and seizings, but we did not consider such repetitions as a beginning of the attack. If a hamster managed to grab the insect with its teeth (bite), it handled it with both forepaws. A successful capture of the insect with their paws was necessarily followed by a bite preceding eating (see details: [33]). The results are presented in Fig. 2. *A. eversmanni* began an attack with seizing with its forepaws less often than other hamsters. *P. sungorus* preferred to initiate an attack with seizing with the forepaws and did so more often than other species, except for *P. roborovskii*. The proportions of attacks that began with seizing with the forepaws were similar in *A. curtatus*, *P. campbelli*, and *P. roborovskii*.

**Fig. 3:**
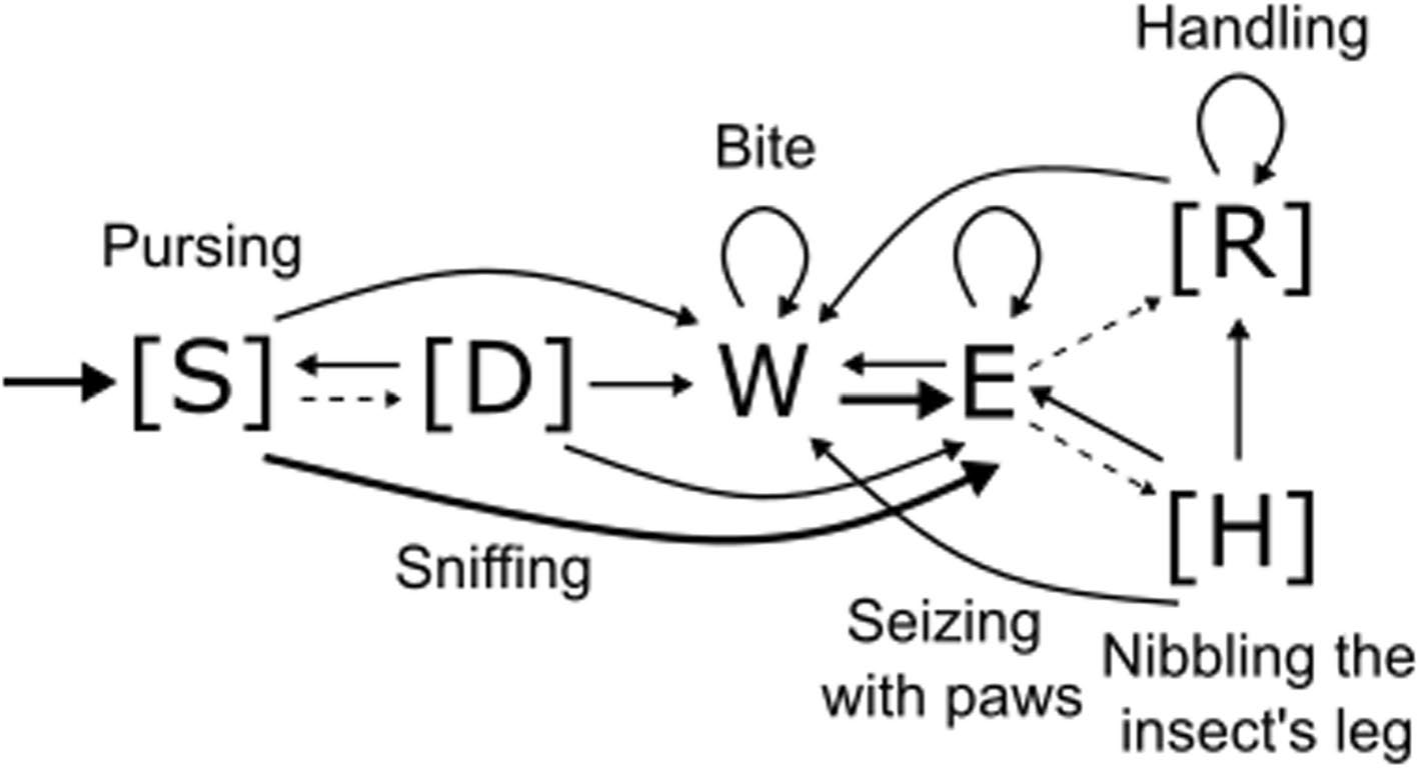
The hunting scheme of *P. roborovskii*. In the scheme, thick lines indicate highly stable relationships between behavioral elements (*p* ≥ 0.5). Plain black lines indicate stable relationships (0.2 ≤ *p* < 0.5). Thin dotted lines denote some unstable relationships between elements, which are important for pattern implementation (*p* < 0.2). In this case, the *p* character means the transition probability, not the statistical significance level. Auxiliary behavioral elements are shown in parentheses

Figure 3 presents a schematic of the hunting sequence of *P. roborovskii* based on the transition probability matrix between behavioral elements. Hunting began with approaching the prey (“S”), which could be followed by sniffing (“D”), or the animal immediately initiated an attack. Regardless of the attack mode, prey was always captured by seizing with paws (“E”), and then, *P. roborovskii* manipulated the prey by handling (“R”) or nibbling insect’s legs (“H”). Then, the hamster bit the prey one or several times (“W”) and ate it.

### Complexities of hunting sequences

**Fig. 4:**
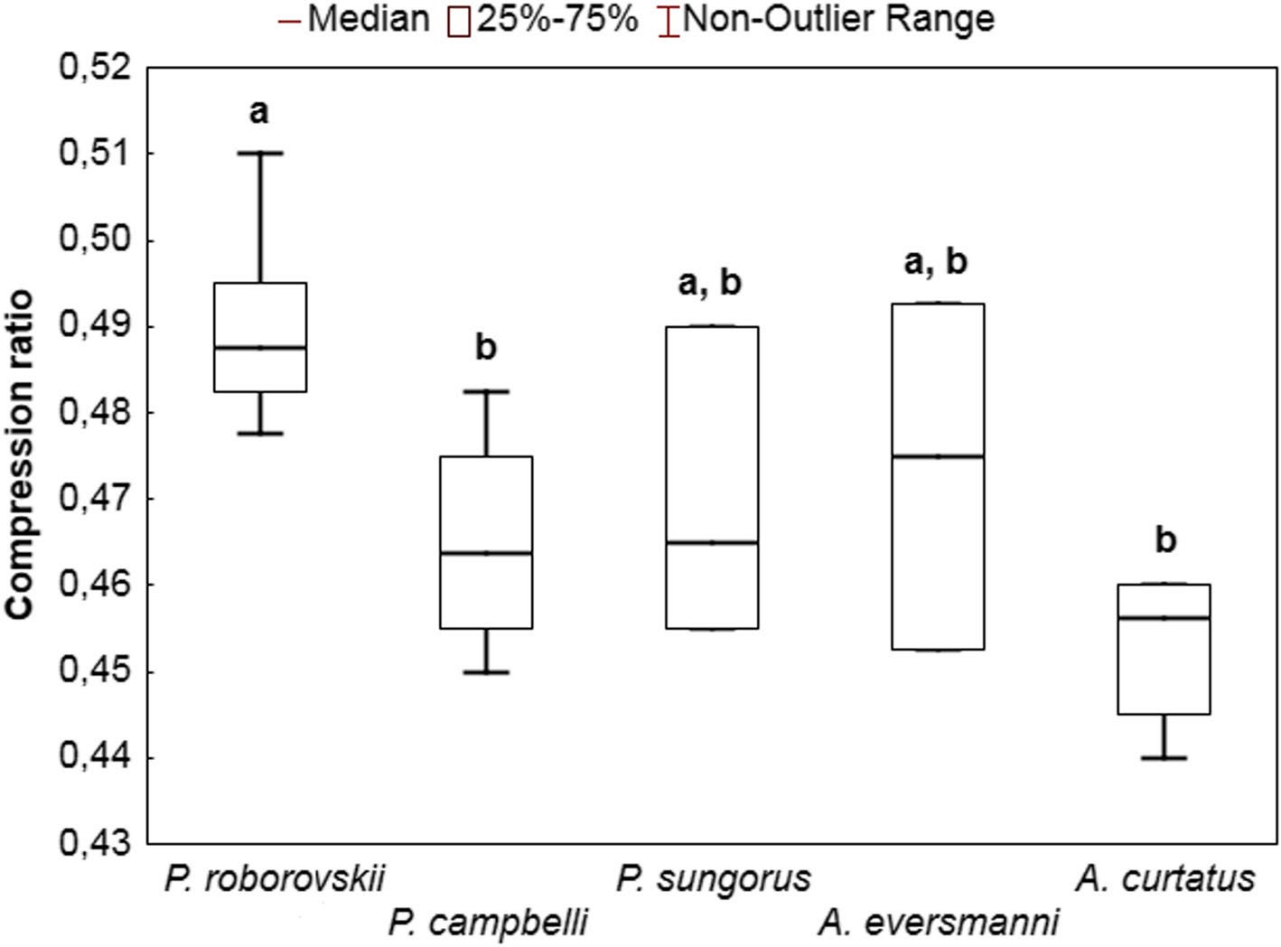
Differences between the average compression ratios of behavioral sequences in the studied hamsters. The same letters (**a** and **b**) indicate no significant differences between the average values of compression ratios in different species (*p* < 0.01) according to *U*-test

Compression of hunting sequences in *P. roborovskii* was weaker (the average compression ratio was 0.491), i.e. it was more complex than that in *P. campbelli* (0.465) and *A. curtatus* (0.457) (Fig. 4).

To answer the question of what are the features of sequences responsible for their complexity, we calculated the Pearson correlation coefficient for the degree of compression and the proportions of different behavioral elements. The proportion of noise elements correlated positively with the degree of compression (*r* = 0.77, *p* < 0.01); on the contrary, the proportion of auxiliary elements correlated negatively (*r* = –0.44, *p* < 0.05). There was no correlation between the degree of compression and the proportion of key elements (*r* = 0.2, NS). Sequence length also did not correlate with the data-compression ratio (*r* = 0.31, NS).

### Homogeneity of Hunting Sequences

**Fig. 5:**
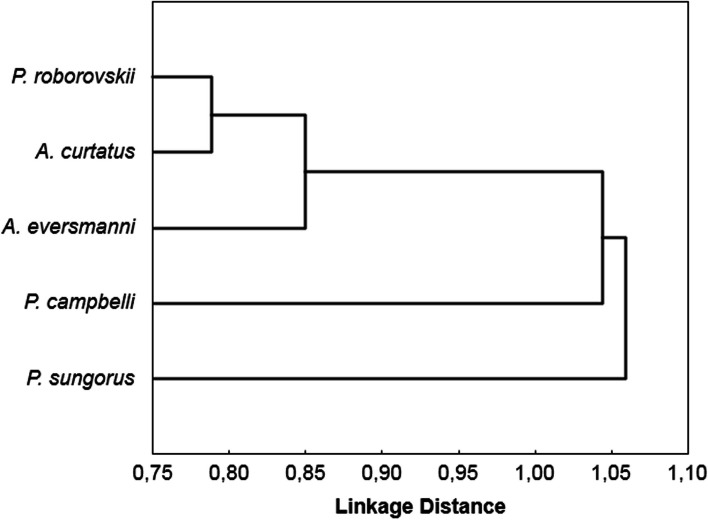
A dendrogram of similarity between hunting behaviors in the species studied, based on the association coefficients from Table 2A (see Supplementary)

To reveal the possible specificity in hunting modes, we compared the association coefficients between parameters of hunting patterns in five hamster species. The result is presented as a dendrogram in Fig. 5. The hunting sequence in *P. roborovskii* was close to that in *Allocricetulus* species and differed significantly from that in other *Phodopus* representatives (Table 3). Hunting sequences in *A. eversmanni* and *P. sungorus* also differed significantly from each other.

**Table 3:** The values of Fisher’s exact test for the 2 × 2 matrices (* *p* < 0.05)

Species	*P. roborovskii*	*P. campbelli*	*P. sungorus*	*A. eversmanni*	*A. curtatus*
*P. roborovskii*	X	0.029*	0.018*	1	1
*P. campbelli*		X	0.2	1	0.23
*P. sungorus*			X	0.015*	0.1
*A. eversmanni*				X	0.15
*A. curtatus*					X

To assess whether the association coefficient is significant for each of the 2 × 2 matrices, we calculated the value of Fisher’s exact criterion (Table 3).

## Discussion

In our study, we investigate possible factors influencing the evolution of non-predatory rodent species’ hunting behavior based on patterns’ structural characteristics. We applied quantitative analysis and estimated the complexity and homogeneity of behavioral sequences in various species to test the hypotheses about the possible roles of phylogenetic differences or ecological influences in the divergence of species’ predatory lifestyles in hamsters. Using the compression-based method, we show the degree of proximity and variability (complexity) of hunting patterns in the species studied. Together with data from quantitative analysis, this allows us to presume the paths of evolutionary change in hunting behavior.

Having studied the hunting behavior in naïve laboratory-born *P. roborovskii* hamsters, we found its optional pattern to be the same as that in the previously studied *Phodopus* representatives [26] and similar to that in other *Muroidea* species [7, 16, 33]. We found an obligate behavior, similar to that in carnivorous grasshopper mice [13, 16], in members of the *Allocricetulus* genus [15, 26]. Since the *P. roborovskii* species and *Phodopus* genus are ancient, we may suggest that the initial hunting behavior in Palearctic hamsters (*Cricetinae*) is optional. The hunting pattern in the young *Allocricetulus* group is obligate; this is a more progressive trait that brings them closer to true predators, grasshopper mice *Onychomys*.

When discussing the hunting behavior of non-predatory hamsters, we cannot speculate on the effectiveness or success of hunting because, despite differences in the specifics of manifestation (obligate/facultative), active hunting behavior is not necessary for the survival of these species. But when conditions deteriorate, insect hunting can be an advantage. Data from stomach contents, cheek pouches [19, 21] and the digestive system [20] indicate the presence of animal food in the diet of the species studied. However, animal food only sometimes involves active hunting: hamsters may find dead insects or small vertebrates or pick them up when they are less mobile (after moulting, during morning dew, etc. No studies of active hunting behavior in non-predatory rodents in natural conditions exist. The rate of successful hunting in the studied species varied from 46 to 70%. This is a high hunting success rate. Under similar conditions, similar rates were observed in specialized predators: 62% in the insectivorous common shrew (*Sorex araneus*, Linnaeus, 1758) [15] and 70–90%, in the southern grasshopper mouse *O. torridus* [2, 13]. By comparison, the successful hunting rate in large predatory mammals under natural conditions is usually about 50% [37]. Note that this characteristic can vary widely even among specialized species. For example, in the cheetah (*Acinonyx jubatus*, Schreber, 1775), successful prey capture was observed in 50% of cases [38], and the successful hunting rate in this species was only 26% according to Wilson et al. [39].

The repertoire of hunting behavioral elements in the studied hamster species was similar to that in the previously described representatives of *Muroidea* [9, 33]. In general, the hunting sequence in hamsters involved grasping prey with teeth and seizing it with forepaws (key elements), processing the prey (auxiliary elements), and actions not related to capture (noise elements). The lowest proportion of noise elements in *A. curtatus* allows us to assess it as the most ‘focused’ hunter on the prey. Analysis of the composition of behavioral elements showed that the evolutionarily “young” *Allocricetulus* species with obligate hunting behavior were characterized by a higher proportion of auxiliary elements and a lower proportion of key elements in the sequence compared with those in the representatives of the more ancient genus *Phodopus*. This suggests that the main evolutionary changes in hunting sequences in *Allocricetulus* and *Phodopus* are associated with the ratio of auxiliary and key elements.

Ideas about the evolutionary pathways of hunting behavior in rodents may be inferred from analyzing the features of attack on prey. As mentioned above, in predatory terrestrial vertebrates, the beginning of an attack on prey using the jaws (grasping with teeth) is considered to be more evolutionarily ancient, whereas seizing prey with the forepaws is believed to be a more progressive trait [17]. A similar concept was proposed for rodents by Langley [3, 16]. Quantitative analysis of the hunting behavior in hamsters revealed differences in the beginning of an attack among different species. In pairs of sister species, *P. sungorus* initiated an attack with grasping with the forepaws more often (61%) than *P. campbelli* (34%) did; a similar pattern was observed in the pair of *A. curtatus* (47%) and *A. eversmanni* (21%). By comparison, attacks of specialized predatory grasshopper mice *Onychomys* on prey almost always begin with seizing with the forepaws [2, 13]. However, in the studied representatives of *Murinae* and *Arvicolinae*, attacks in most cases began with a bite [9, 33, 40]; in two *Gerbillinae* species, the Mongolian gerbil (*Meriones unguiculatus*, Milne-Edwards, 1867) and the fat-tailed gerbil (*Pachyuromys duprasi*, Lataste, 1880), successful attacks in 8.6% and 31.6% of cases, respectively, involved only seizing with the forepaws. Hence, all the studied species demonstrate, although to varying degrees, an advanced mode of prey capture, which may be considered an adaptation to mobile insect hunting, and this brings their hunting behavior closer to that of specialized predatory hamsters *Onychomys*.

Quantitative assessment of complexity revealed that the degree of hunting sequence compression in *P. roborovskii* was lower than that in *P. campbelli* and *A. curtatus*. This suggests that the hunting behavior of *P. roborovskii* is more variable. On the contrary, behavioral sequences in *P. campbelli* and *A. curtatus* are characterized by greater order and predictability of behavior during hunting. Correlation analysis revealed that an increase in the proportion of auxiliary elements reduced the complexity of hunting sequences, whereas an increase in the proportion of noise elements resulted in complexity rise. Auxiliary elements in the pattern are associated with processing of prey, while noise elements are not associated with the hunting process at all. Auxiliary elements appear to avoid random (“noise”) or repetitive hunting behavior, which makes the hunting behavior more orderly and predictable (i.e. less complex). For example, by having made several handling movements (“R”), the animal more reliably holds prey with its forepaws and is less likely to miss it, thereby avoiding the need for repetitive pursuit and capture.

Previously, we identified hunting behavioral patterns and used them to describe hunting tactics in two *Phodopus* and *Allocricetulus* species [26]. In this study, we developed a scheme for *P. roborovskii*, which turned out to be similar to that for *Allocricetulus*. We define the hunting tactics as a set of actions that lead to the most rapid damaging of the prey, followed by its eating. Hunting sequences in *Allocricetulus* and *P. roborovskii* involve repetitive handling of prey (“R”) followed by one or more bites (“W”). A distinctive feature of *P. campbelli*’s hunting was prey immobilization tactics by nibbling its legs: the element (“H”) was more often found in hunting sequences of this species (Table 2). This also makes the sequence more predictable (less complex): the likelihood of repetitive pursuit and capture of prey was reduced. The hunting tactics of *P. sungorus* lacked any distinctive features: capture, minimal manipulation, and immediate eating of prey. In general, this is consistent with the identified differences in the ratio between proportions of the auxiliary and other elements in hunting sequences and the assumption of their role in the development of hunting tactics. We suggest that hunting tactics do not underlie hunting behavioral complexity in hamsters: similar tactics were formed in *A. curtatus* and *P. roborovskii*, which differed in the complexity of hunting sequences.

In our recent paper [27], we applied the method of homogeneity of hunting sequences to reveal differences in hunting modes in nine rodent species. We found four hamster species within a separate cluster. In this study, we showed essential differences when *P. roborovskii* was added. Optional hunting behavior in *P. roborovskii* displayed similarities with the obligate patterns in *Allocricetulus* species; therefore, it turned out to be the most advanced hunter among members of the *Phodopus* genus (Fig. 5). Differences in hunting sequences among *Phodopus* representatives suggest that the hunting behavior of these species, despite its optional mode, was subject to selection during species splitting within the genus.

To imagine the possible evolutionary pathways of the hunting behavior in hamsters, we compared the data on homogeneity of behavior sequences (Fig. 5) and the molecular clock analysis data [22, 25]. The hunting behavior of *P. roborovskii*, which is believed to be the most ancient part of the *Phodopus* branch, was close to that of a younger *Allocricetulus* group and significantly differed from the two sister *Phodopus* species. This suggests that the differences among *P. campbelli*, *P. sungorus*, and *P. roborovskii* in the modes of prey processing have arisen relatively recently. The ancestors common to both groups probably had a reasonably progressive hunting sequence, which might begin with capturing prey with both the teeth and the forepaws. Further evolution raised the priority of starting an attack with the forepaws. In *A. eversmanni*, the hunting sequence has most likely remained in its original, more ancient form (the beginning of an attack with a bite predominates), or there has been a secondary shift in the beginning of the attack towards grasping with the teeth. The beginning of the *A. eversmani*’s attack with a bite probably results from adaptation to hunting vertebrate prey. This species is more aggressive than *A. curtatus*. In the experiments with a pair of conspecifics placed in the same area, *A. eversmanni* behaved like hunting predators; in some cases, if the researcher failed to separate animals in time, one of the animals delivered a quick killing bite to the parietal area of the victim’s skull [41]. The carnivorous hamster *O. leucogaster* behaves similarly when interacting with other rodent species [42], and *O. torridus*; when hunting horned lizards, it bites prey in the head in 40% of cases [43]. An analysis of the stomach contents in the *A. eversmanni* indicates the presence of many remains of various small vertebrates [19], which indirectly confirms the assumption of a more carnivorous diet of the species. Vorontsov [20] noted that the studied species’ protein-lipoid type of nutrition characteristic suggests the need for animal-protein food in the diet. In addition to providing lipids, ecological relevance, and proteins, insects can also be a source of moisture, vital for species living in arid landscapes. We suggest that the hunting sequences in *P. roborovskii* and *A. curtatus* evolved convergently as an adaptation to the desert habitat. The modern ranges of these species overlap [22]. According to our observations, *Phodopus* and *Allocricetulus* hamsters in arid habitats often shift from seeds to larvae and adult insects. This issue requires further research.

In conclusion, our results did not reveal the role of phylogenetic differences in the divergence of species’ predatory lifestyles. We suggest that ecological conditions are the main factors in speciating the hunting behavior in hamsters.

### Supplementary Information

Supplementary Material 1.

## Data Availability

The datasets during and/or analyzed during the current study available from the corresponding author on reasonable request.
