# Supplementary material for: Comparative analysis of optional hunting behavior in Cricetinae hamsters using the data compression approach
Source: Front Zool. 2024 Jul 15;21:19. doi: 10.1186/s12983-024-00540-4 (PMC11247907; doi:10.1186/s12983-024-00540-4)
Supplement: Supplementary file 1 — Supplementary Material 1. [file 12983_2024_540_MOESM1_ESM.docx]

**Supplementary**

*Preparation of Sequences / Data Preparation*

For comparative assessment of complexity, we divided the raw behavioral data files (Table 1A, column 2) into multiple sampled data text files, 400 bytes in size each (Table 1A, column 4). We obtained different numbers of sample data files because the lengths and numbers of behavioral sequences and, correspondingly, the sizes of the raw behavioral data files were different for each species (Table 1A, column 3).

**Table 1A** The volumes of data obtained

| **Species** | **Sizes of Raw Data Text Files (bytes)** | **Numbers of Sequences in Raw Data Text Files** | **Number of Sample Data Files Obtained** | **Sizes of the First Parts of the Raw Data Text Files (Bytes)** | **Number of the Sample Files Obtained from Seconds parts** |
| --- | --- | --- | --- | --- | --- |
| *P. roborovskii* | 2934 | 39 | 5 | 1455 | 4 |
| *P. campbelli* | 1715 | 43 | 4 | 801 | 4 |
| *P. sungorus* | 1585 | 76 | 3 | 795 | 6 |
| *A. eversmanni* | 1463 | 60 | 3 | 731 | 5 |
| *A. curtatus* | 2814 | 115 | 6 | 1407 | 9 |

For homogeneity testing, we divided the raw behavioral data files into two approximately equal parts (Table 1A, column 5). The first file containing half of the data was used as a whole for further calculations. The second file was divided into several fragments (sample text files) using a special program, each sized 120 bytes (Table 1A, column 6). We then added the sequences of each sample text file to the first half of the files. Therefore, we got augmented text files, their number corresponding to the number of sample text files multiplied by the number of types (a total of 140). All the received augmented text files were compressed by an archiver. Further comparisons were carried out in pairs. The number of cases was calculated in which the augmented text file archive with sequences of only one species was smaller than the archive with an augmented text file, where the first half was of the same species and the sample text files were of a different species. The results were input into 2 × 2 matrices. A total of ten matrices were obtained. For each of them, the association coefficient was calculated [35] (details in: [27]). By applying Fisher’s exact test to the 2 × 2 matrices, we can evaluate the significance of differences between the association coefficients. To obtain a dendrogram of similarity, we placed all the obtained values of the association coefficients in the *K* × *K* matrix (where *K* is the number of species) symmetrically with respect to the diagonal, and the clustering method was then applied to the resulting matrix (Table 2A).

**Table 2A** Volumes of coefficients of association for the 2 × 2 matrices

| **Species** | ***P. roborovskii*** | ***P. campbelli*** | ***P. sungorus*** | ***A. eversmanni*** | ***A. curtatus*** |
| --- | --- | --- | --- | --- | --- |
| *P. roborovskii* | 0 | 1 | 0.79 | 0 | 0.09 |
| *P. campbelli* | 1 | 0 | 0.53 | 0 | 0.44 |
| *P. sungorus* | 0.79 | 0.53 | 0 | 0.83 | 0.49 |
| *A. eversmanni* | 0 | 0 | 0.83 | 0 | 0.45 |
| *A. curtatus* | 0.09 | 0.44 | 0.49 | 0.45 | 0 |
